# Colorless Tincture of Iodine

**Published:** 1873-06

**Authors:** 


*Colorless Tincture of Iodine.*—The greatest objection to the officinal tincture of iodine so useful in alveolar periostetis, as to be regarded by some practitioners as a specific, is the discoloration it causes when externally applied. The *Medical & Surgical Reporter* gives a formula for a colorless tincture of iodine, which is said to be convenient and efficacious. It as follows:

℞. Tincture of iodine,

Pure glycerine, aa ℥j.

Sulphite of Soda, ʒj.

Rub the salt to a powder in a small mortar, and add the glycerine gradually; then pour in the tincture of iodine, and triturate gently until a solution is effected, and the mixture assumes an amber color. It is asserted that the properties of iodine are increased by the addition of the sulphite of soda, and that the glycerine enhances the value and convenience of the preparation for local application.

